# Assessment of the Structural Integrity of the Portal Crane Elements After Long-Term Operation

**DOI:** 10.3390/ma17246133

**Published:** 2024-12-15

**Authors:** Ihor Dzioba, Olha Zvirko, Robert Pała, Oleksandr Oliynyk

**Affiliations:** 1 Faculty of Mechatronics and Mechanical Engineering, Kielce University of Technology, Av. 1000-An. of Polish State 7, 25-314 Kielce, Poland; rpala@tu.kielce.pl; 2 Department of Diagnostics of Materials Corrosion-Hydrogen Degradation, Karpenko Physico-Mechanical Institute of the NAS of Ukraine, 5 Naukova St., 79060 Lviv, Ukraine; olha.zvirko@gmail.com; 3 Department of Lifting and Transport Machines and Engineering of Port Technological Equipment, Odessa National Maritime University, 34, Mechnikova St., 65029 Odesa, Ukraine; dialab.sec@gmail.com

**Keywords:** portal crane, microstructure, long-term operation, mechanical properties, fracture toughness, FAD (FITNET) assessment procedure

## Abstract

Evaluating the current technical condition and residual life of structures that may have reached or exceeded the end of their design life is a challenging issue in many industrial sectors. This paper focuses on the assessment of the structural integrity of structural elements of a seaport portal crane after operation for about 33 years. Test specimens were extracted from two crane elements, a jib (element A) as the most seriously loaded unit and, for comparison, a boom (element B) as the less loaded one, and machined in two different orientations, longitudinal and transversal. Both crane elements were made of a low-carbon rolled steel. The analysis was performed according to the FAD approach of FITNET Procedures. The basic level of analysis was used. For this purpose, the strength and fracture toughness characteristics of the steel from tested elements were determined experimentally. The strength and plasticity properties of the steel of both elements were similar. At the same time, the fracture toughness characteristics differ for the elements A and B and for the longitudinal and transverse specimen directions. The results of the FAD (FITNET) analysis performed for the elements containing a central crack loaded by tensile stress showed that they are not susceptible to failure. The relative length of cracks for which the critical state will be reached is also estimated.

## 1. Introduction

Evaluating the current technical condition and residual life of structures that have reached or exceeded their design life presents significant challenges across various industrial sectors [1]. This assessment is critical for ensuring safety and reliability, particularly in critical infrastructure such as bridges and cranes, which are often subjected to fatigue and deterioration over time [1,2,3,4,5,6,7,8]. Metal structures and equipment during long-term operation are exposed to degradation of the material’s mechanical properties, which reduces their performance characteristics. The material degradation of engineering objects is influenced by various factors—temperature, the working environment (corrosive environment, hydrogen influence), and long-term cyclic or static mechanical loads. Therefore, it is important to periodically test engineering elements to determine the current state of material properties and assess whether they are suitable for further operation [1,2,3,4,5,6,7,8]. To this end, testing is carried out directly on the object under analysis, using penetrant, ultrasonic, and non-destructive methods, among others [9]. More reliable assessments can be obtained if taking samples and performing strength and fracture toughness tests is also possible. However, it is not always possible to collect enough material from the structure in service to perform these tests. In most cases, investigators must assess the service life of the structure while being limited by the material for testing.

In the case of long-term exposure of structures or their components to elevated temperatures (over 450 °C), such as components of district heating pipelines at heat and power plants, significant changes in the microstructure, phase composition, and shape of carbides occur in the steel [10,11], leading to a deterioration of the mechanical properties, mainly a reduction in fracture toughness characteristics [12,13,14]. While no microstructural changes are observed in the material during the long-term operation of structures at environmental temperatures, a reduction in material properties is still noted. Various studies attribute the decrease in characteristics to the combined effects of stress, corrosion, and hydrogen on the metal [15,16,17]. In gas or oil pipelines operated at ambient temperature, the influence of hydrogen on material degradation is indisputable [18,19]. Similarly, in crane or bridge structures operated under the mutual action of cyclic loading and environmental impact, steels are exposed to hydrogen-assisted degradation as a result of hydrogen evolution under atmospheric corrosion when protective coatings are damaged [20,21]. In such a case the degradation of material properties primarily affects ductility and fracture toughness, while the strength characteristics remain relatively unchanged [19,22,23,24,25]. Structural steels produced by rolling typically feature a banded microstructure, resulting in anisotropic mechanical behaviour [23,24,26,27,28,29,30], which can be noticeably amplified due to long-term operation [24,26].

Therefore, regular assessments of the technical condition of long-term operated metal structures are required. For example, the orders of the State Administration of Market Surveillance of China indicate a period of 15 years of crane operation as necessary to carry out strength tests and reveal any degradation of crane characteristics for safe continued operation [31]. Similar requirements for the safe operation of cranes are made in other countries [32,33].

Numerical assessment methods involving modelling and simulation of the structure’s operation are increasingly used in safety analyses. FEM analysis is used for the strength and static stiffness of the gantry cranes [34], which is helpful for choosing proper reinforcement measures and can be used as a basis for the reconstruction or strengthening of cranes. Domazet et al. [35] conducted a fatigue damage analysis and outlined a repair procedure following the detection of cracks on a 250 kN portal crane. Numerical analysis results and strain gauge measurements identified the source of crack propagation as high-stress concentrations as a result of inappropriate design. Based on FEM models, proper measures were chosen (adding stiffeners) in order to reduce stress concentrations and, consequently, critical areas of the crane were redesigned, and subsequent measurements showed no new cracks were found after two years of service [35]. In the study by Chen and He [36], a portal crane with a rated capacity of 12 tons was subjected to a numerical model after more than 50 years of service. The numerical model used data from the measurement of the true condition of the crane (taking into account any reduction in material thickness). The maximum levels of effective stresses were determined to be approximately 130 MPa at the most loaded point during crane operation. In the work of Nemchuk and Nesterov [22,23], similar results were obtained by numerical analysis of crane operation. In addition, strain gauge measurements were taken at the most loaded parts. Nevertheless, data on the structural integrity assessment of structural elements of portal cranes are limited. A conservative assessment during the safety analysis of structures can be performed using the FAD approach of FITNET Procedures [37]. It can be applied to different welded and non-welded metallic structures operated under various operating conditions. Thus, using the FITNET procedures according to the Failure-Assessment-Diagram (FAD) approach, it was demonstrated in [38] that the base metal of gas transit pipelines both in an as-received state and after 40 years of operation is more susceptible to fracture due to reaching the critical stressed state than the weld metal.

Considering that degradation of the material’s mechanical properties occurs during the long-term operation of port crane elements under environmental conditions and varying loads, this paper is focused on the evaluation of the current condition of the material. The assessment method using the FAD approach presented in FITNET materials [37] was applied to the analysis of structural integrity elements of a seaport portal crane. The evaluation was carried out at the basic level, where it is necessary to know the basic strength characteristics and fracture toughness of the material.

## 2. Materials and Methods

**Figure 1:**
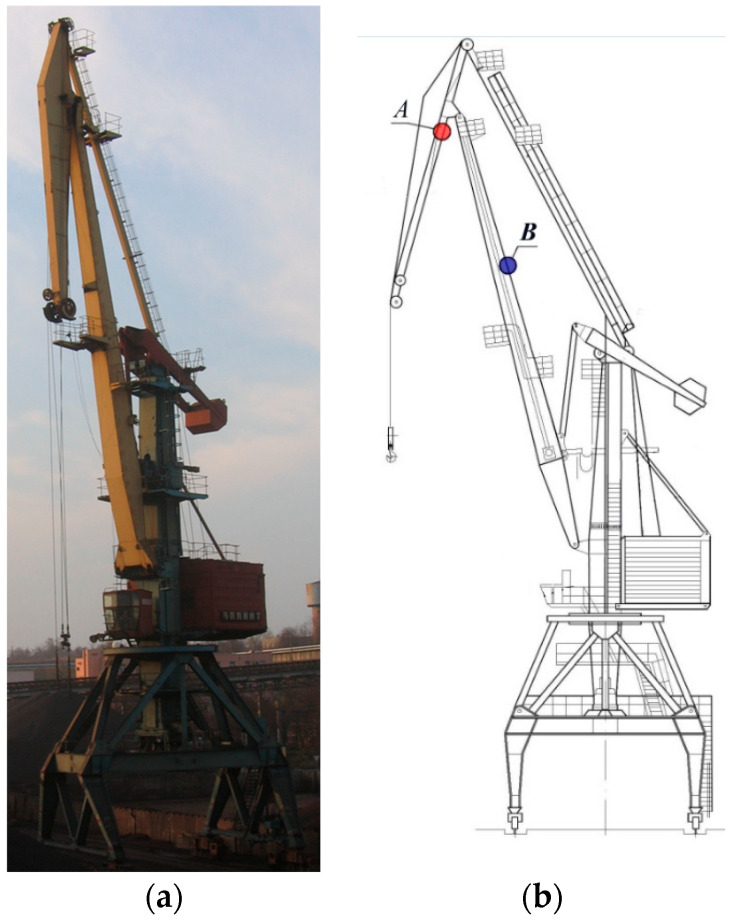
(**a**) Photo of a portal crane. (**b**) Scheme with identification of the zones where the material was analysed.

This study analysed the condition of material taken from two different elements of a portal crane (Figure 1a), which was in service for about 33 years. During the long-term operation of the crane, elements A (a jib, thickness 16 mm) and B (a boom, thickness 12 mm) were cyclically subjected to stresses of different levels (Figure 1b). On the basis of numerical calculations, the most loaded elements of the crane were determined, in which the range of stresses, Δ*σ*_ref_, was then determined using strain gauge measurements: in element A (a jib), the stress range was within 0–130 MPa, while for element B it was 0–55 MPa [24].

**Figure 2:**
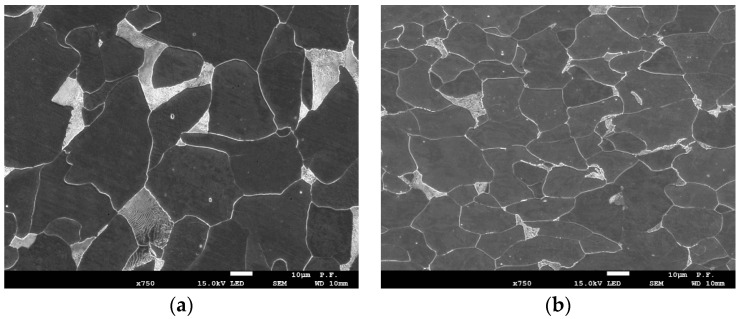
(**a**) microstructure of the steel from element A; (**b**) microstructure of the steel from element B.

Although the crane elements are made of the same grade of steel (see Table 1), and both have a ferritic–pearlitic type of microstructure, some differences in their microstructure were observed. For the material of element A, the grains of ferrite and pearlite are larger compared to the steel of element B (Figure 2a,b).

## 3. Results: Experimental Studies—Determination of the Characteristics of the Strength and Fracture Toughness of the Materials

**Figure 3:**
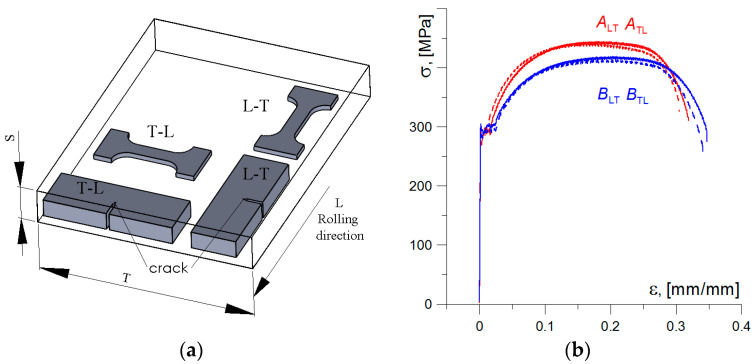
(**a**) Scheme the specimens cutting from the plate relative to the rolling direction. (**b**) Stress–strain diagrams of the tested specimens.

Specimens for uniaxial tensile and fracture toughness tests were made from cuts taken of the crane components marked as A and B (Figure 1b). All test specimens were cut from elements A and B in longitudinal (specimen codes—A_LT_ and B_LT_) and transverse (specimen codes—A_TL_ and B_TL_) orientations according to rolling direction (Figure 3a). For specimens of longitudinal orientation, the plane of fracture propagation was perpendicular, and for specimens of transverse orientation, the plane of fracture was parallel to the rolling direction when they were tested.

Due to the material limitation, only three specimens were tested in tests to determine strength characteristics and fracture toughness characteristics. The results presented in Table 2 and Table 3 correspond to the minimum values of the characteristics obtained during the tests.

The experimental tests were carried out under ambient conditions on a Zwick/UTS-100 (Ulm, Germany) electrodynamic testing machine equipped with an automated control and data recording system. Uniaxial tensile tests were carried out according to ASTM [39] and ISO [40] standards. Fracture toughness tests were performed according to ASTM standards using Single Edge Notch in Bending (SENB) type specimens [41]. During uniaxial tensile tests, an extensometer was used to measure the elongation of the measured part of the specimens. In the case of determining fracture toughness, two extensometers were used—an extensometer to determine the specimen’s deflection and an extensometer to measure the crack opening—COD.

### 3.1. Uniaxial Tensile Test Results

At the uniaxial tensile test, specimens with a rectangular cross-section of 5.0 × 3.5 mm^2^ and a gauge length of 20.0 mm were tested. Dashes were made on the gauge section of the specimens at 2.0 mm intervals. During the tensile test, video recording was used until the specimen failed, allowing strain levels to be determined at each point of testing across the entire gauge section and also at the most elongated sections, where a local constriction—the neck was formed. The nominal values of strength (lower and upper yield stress σ_LYS_ and σ_HYS_, respectively, and ultimate strength *σ*_UTS_) and plasticity (uniform and non-uniform relative elongation *EL*_U_ and *EL*_NU,_ respectively, and total elongation *EL*_F_, and reduction in area, *RA*) characteristics of the tested specimens are presented in Table 2, and the stress–strain diagrams, *σ*_n_–*ε*_n_, are shown in Figure 3b.

The presented results show that the steel of both investigated elements A and B has a clear yield point with a similar level of value. Some differences were noted for the ***σ*_UTS_** characteristic—the values for material A are higher compared to B. On the other hand, material B is characterised by higher plasticity than A. When comparing the stress–strain curves of specimens cut in the longitudinal and transverse directions, it is difficult to identify any significant differences in the values of strength characteristics. However, slightly higher levels of plasticity characteristics were obtained on specimens of longitudinal orientation (see Table 2).

**Figure 4:**
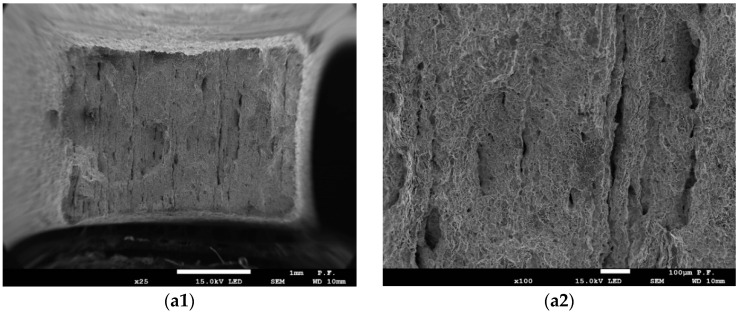

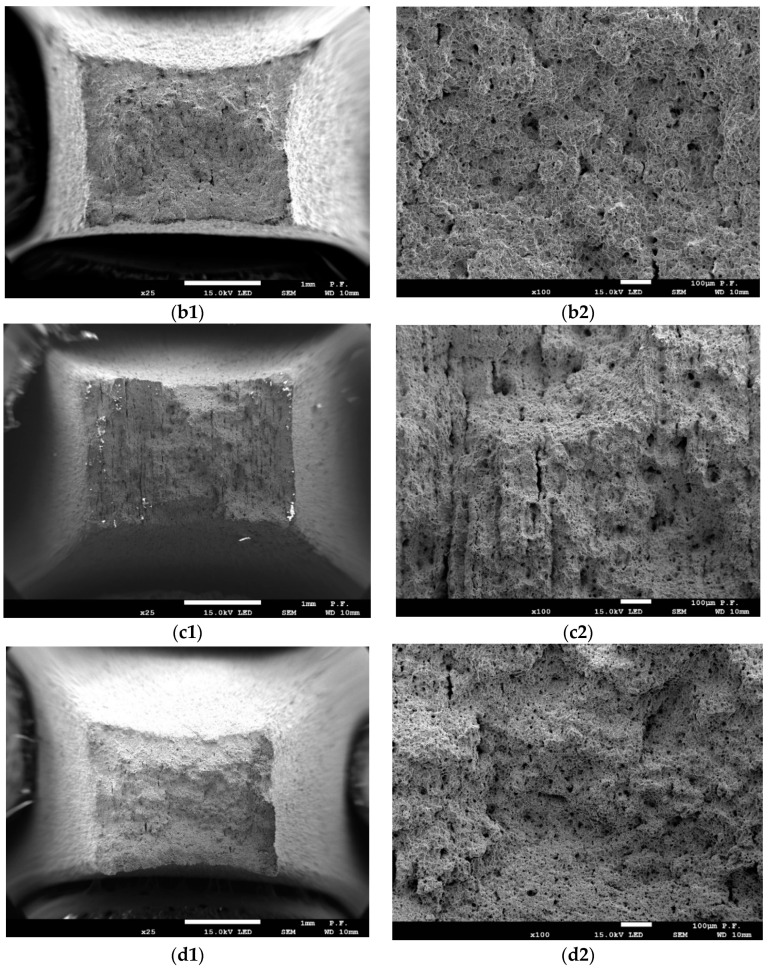
Typical fracture surfaces of the tested tensile specimens: (**a1**,**a2**) A_TL_; (**b1**,**b2**) A_LT_; (**c1**,**c2**) B_TL_; (**d1,d2**) B_LT_; (1—×25; 2—×100).

Fracture surfaces of tensile specimens for the studied steel are illustrated in Figure 4. The macroanalysis revealed a clear arrangement of non-metallic inclusion bands on the fracture surfaces. Numerous longitudinal microcracks (delamination cracks) formed as a result of the chipping of these non-metallic inclusions (Figure 4a1,a2,c1,c2) are detected at the fracture surface of specimens cut off transversely A_TL_ and B_TL_. At the same time, the number of such defects is significantly lower (Figure 4b1,b2,d1,d2) at the fracture surface of longitudinal specimens A_LT_ and B_LT_. Comparing the fracture relief of specimens, one can see that the material of element A has a greater number of defects than that of element B.

### 3.2. Determination of the Fracture Toughness Characteristics

Since the tested materials have a high level of plasticity, the critical fracture toughness value *J*_C_ was determined on the basis of the *J*_R_ curve prepared according to the recommendations of the ASTM standard [41]. The fracture toughness characteristics were determined during the testing of Single Edge Notch in Bending (SENB) specimens with the following dimensions: *B* = 12 mm; *W* = 24 mm; *S* = 96 mm; *a*_0_/*W* = 0.5. The thickness of the tested specimens depended on the thickness of the plates of the tested crane elements. During the preliminary tests, it turned out that it does not meet the requirement of the standard according to *B* (*B* ≥ 25 × *J*_C_/*σ*_YS_). Therefore, on the side surfaces of the SENB specimens, side grooves with a depth of 1.0 mm were cut. This procedure enables obtaining a stress distribution in the plane before the crack tip similar to the state of plane strain, and the thickness of such a sample is assumed to be *B*_e_ = (*B* × *B*_n_)^1/2^, where *B*_n_—is the width of a specimen with side grooves.

**Figure 5:**
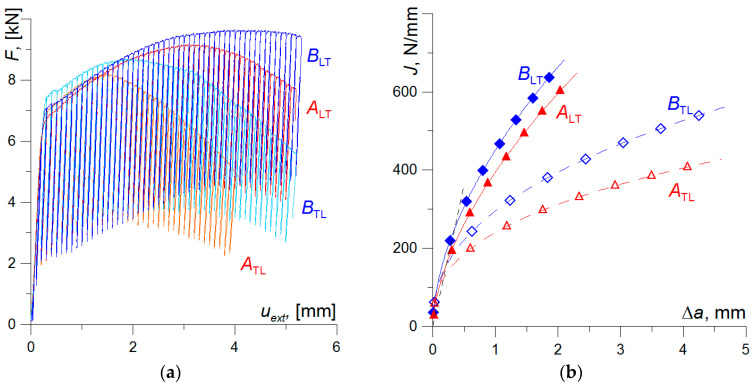
The load—displacement, *F*—*u*_ext_, diagrams (**a**), and *J*_R_ resistance curves (**b**) for the tested specimens.

Figure 5 shows the load versus deflection graphs of the specimens, *F*—*u*_ext_, and the graphs of the corresponding *J*_R_ curves. From these graphs, it can be concluded that the fracture toughness depends on the specimen orientation in both elements A and B. The fracture toughness characteristics, the critical *J*_C_ value, and the *J*_R_ curve values are higher for the longitudinal specimens compared to those obtained for the transverse ones (Table 3). Differences in the values of the fracture toughness characteristics are associated with the orientation of non-metallic inclusions along the rolling direction. In the case of testing longitudinal specimens, the plane of growth of the subcritical crack is perpendicular to the rolling direction and the orientation of the inclusions. In transverse specimens, the direction of subcritical crack development and the orientation of inclusions are the same, which facilitates crack propagation due to the tunnelling effect—due to the fracture of brittle particles of non-metallic inclusions and/or due to the loss of cohesion between the inclusions and the ferritic matrix.

The quantity *K*_JC_ in Table 3 is used to represent the fracture toughness values determined as the critical value of a *J* integral in stress intensity factor units and is calculated according to the Formula (1).

(1) $$K_{JC}=\sqrt{\frac{J_{C}E}{1-\nu^{2}}}$$

A comparison of the results obtained for the specimens from elements A and B clearly indicates a higher fracture toughness of the material from element B. The specimens taken from element B showed higher levels of the critical fracture toughness value *J*_C_ and the *J*_R_ curve values compared to element A (Figure 5; Table 3). The tearing modulus of subcritical crack growth resistance: *T*_R_ = (*E*/*σ*_YS_ ^2^) × (*dJ*/*da*) [42,43], determined for the subcritical crack growth Δ*a* = 0.5 mm, is slightly higher for specimens of the appropriate orientation from element B than from A.

Higher impact strength values *KCV* were also obtained for the material from element B than for A. For comparison, the impact strength test results are presented in Table 3. The critical values of fracture toughness *J*_C_ among the tested mechanical characteristics show significantly higher sensitivity to the anisotropy of the material compared to the basic tensile properties. Impact toughness also has a high sensitivity to the microstructure of rolled steel [25].

**Figure 6:**
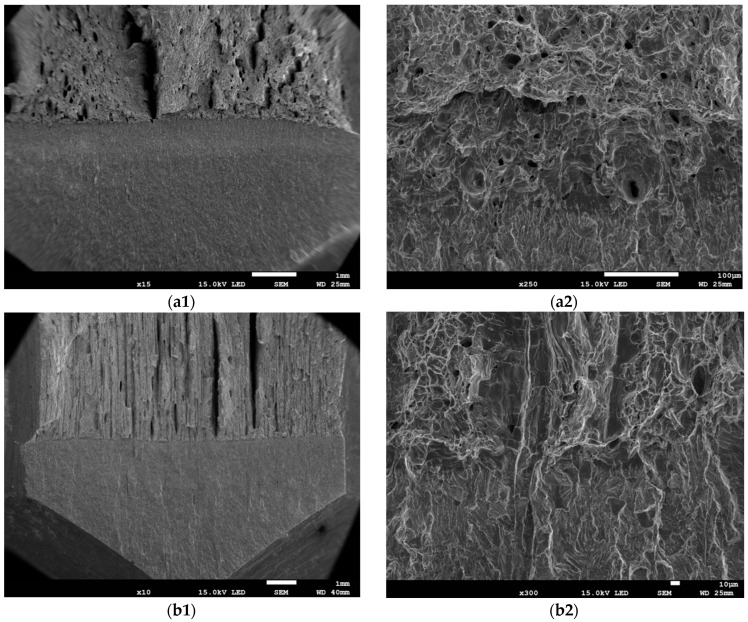

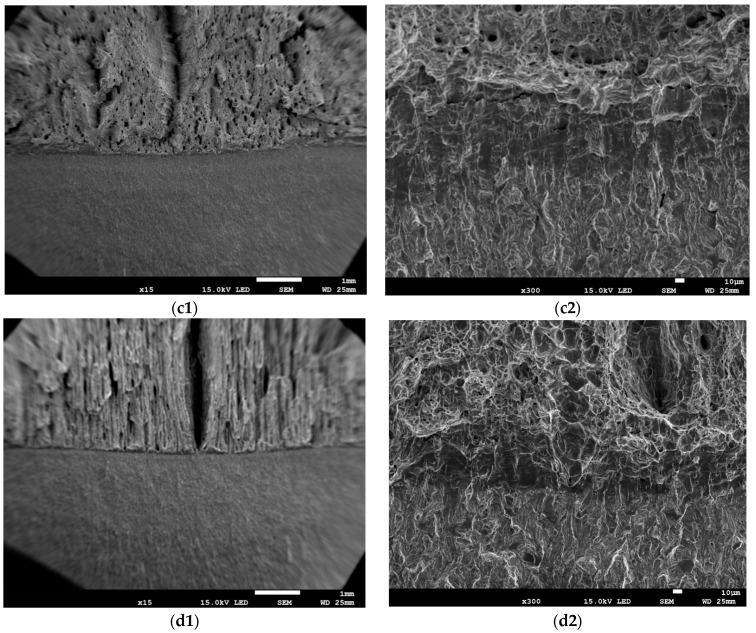
Fracture surfaces of the SENB specimens: (**a1**,**a2**) for A_LT_; (**b1**,**b2**) for A_TL_; (**c1**,**c2**) for B_LT_; (**d1**,**d2**) for B_TL_.

Differences in the fracture morphology of the specimens were observed at the macro (images at ×10, ×15) and micro (images at ×250, ×300) levels (Figure 6). On the fracture surfaces of the longitudinal specimens, the mechanism of void and cavern development dominates, while in the transverse specimens, along with the void growth mechanism, there is a mechanism of delamination of elongated non-metallic inclusion particles from the ferritic matrix. However, in the fracture surfaces of both orientation specimens, the mechanism of void development dominates, which defines the ductile nature of the subcritical crack growth.

### 3.3. In-Service Strength Assessment of Components Based on the FAD Method (FITNET)

The FITNET collections include procedures for assessing the strength of structural components containing crack-type defects.

FITNET procedures [37] were developed based on previously widely used normative documents API579/ASME-FFS [44], BS7910 [45], R6 [46]. They include recommendations for action for assessing the strength of structural elements with crack-type defects in four basic cases: under cyclic loading—the Fatigue module; under the influence of high temperatures—the Creep module; due to the influence of corrosive factors—the Corrosion module; under monotonic or constant loading, when a crack is formed—the Fracture module. The Fracture module presents six levels for the analysis of homogeneous or welded elements made of iron alloys. At higher levels of analysis, extended material data (e.g., true stress–strain relationships) and numerical calculations are required, which allows obtaining results with low safety conservatism. For the assessment at a lower level, knowledge of the basic nominal strength characteristics of the material and fracture toughness (or impact strength) is sufficient. However, the result of the safety assessment is more conservative.

In structural elements, corrosion patterns, large inclusions, and other types of irregularly shaped defects are usually observed. In FITNET procedures [37] it is recommended to present various types of irregularly shaped defects as regular-shaped gaps encompassing these defects. This approach allows the calculation of the values—*K*_I_, *K*_r_, and *L*_r_ and leads to a conservative assessment during the safety analysis.

These procedures allow strength analysis to be carried out at different levels—from simple estimates, which are performed on the basis of knowledge of the strength and fracture toughness characteristics of the materials, to complex ones, where knowledge of the true constitutive relationships of the materials under test and numerical calculations are additionally necessary. The evaluation results obtained at lower levels are always more conservative compared to those at higher levels, where advanced methods are used.

In order to confirm the results obtained above, the present study also evaluated the strength of plates with a central crack made of A or B materials in uniaxial tension. The chosen nature of the loading in the specimen is similar to that realised in the crane elements during its operation. The procedures and formulas suggested for the base (first) level were used.

According to FITNET procedures [37], in order to assess the strength of structural elements containing a crack, it is necessary to know the material strength characteristics *σ*_YS_ and *σ*_UTS_ and the critical value of the stress intensity factor (SIF) *K*_IC_ or *K*_JC_. The *K*_JC_ value is obtained after conversion to SIF units from the experimentally determined critical *J*_C_ values (see relation 1). The critical values after conversion to *K*_JC_ and next to a specimen of reference thickness *K*_mat_ for *B*_ref_ (see relation 2) are presented in Table 4.

(2) $$K_{mat}=K_{min}+\left(K_{JC(B)}-K_{min}\right){\left(\frac{B}{B_{ref}}\right)}^{0.25},$$

where *B* = specimen thickness; *B*_ref_ = 25 mm; *K*_min_ = 20 MPa$\sqrt{m}$.

**Figure 7:**
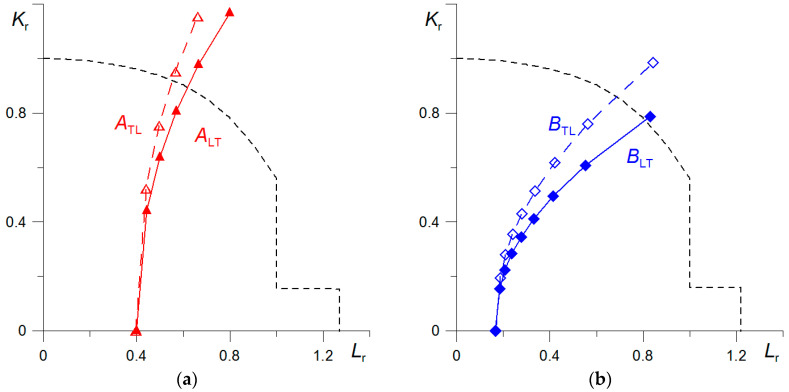
Results of the FAD analysis: (**a**)—for the element A; (**b**)—for the element B.

The material condition was assessed according to the Failure Assessment Diagram (FAD) approach, which compares the state of stresses and strains in the material of the element under analysis with a critical state defined by a failure line, FAD. The FAD line diagrams depend on the strength and plasticity characteristics of the material. The state of the material is expressed by the normalised load *L*_r_ = *σ*_ref_/*σ*_YS_ (or *P*/*P*_0_, where *P*_0_ is the load causing a plastic collapse of the element) and the normalised fracture toughness *K*_r_ = *K*_I_/*K*_mat_. Figure 7 shows the material condition analysis diagrams for elements A and B. The FAD line is drawn according to Formulas (3)–(9), which are recommended for the first level of analysis:

(3) $$K_{r}\left(L_{r}\right)={\left(1+0.5{L_{r}}^{2}\right)}^{-0.5}\left[0.3+0.7exp\left(-\mu {L_{r}}^{6}\right)\right],\;for\;L_{r}\leq 1$$

where $\mu =\min\left[0.001\left(\frac{E}{\sigma_{YS}}\right);0.6\right]$.

The FAD analysis was carried out for a tensile plate of width *W* = 1.0 m with a central crack whose length was varied in the range 0.1·*k*·*W* (*k* = 0; 1; 2;…9). Due to the fact that the load on the plates was given directly by the stress level and the dimensionless relative values *a*/*W* appear in the Formulas (4)–(6), the geometric dimensions of the plate cross-section do not affect the result when calculating *L*_r_ and *K*_r_. The level of loading stress *σ*_ref_ in the analysis carried out was assumed according to the experimentally determined: for the element from material A, *σ*_ref_ = 130 MPa, and for—B, *σ*_ref_ = 55 MPa.

Formulas from the FITNET catalogue [37] are used for the calculations *L*_r_ and *K*_r_:

(4) $$L_{r}=\frac{\sqrt{3}}{2}\frac{\sigma_{ref}}{\sigma_{YS}\left(1-\frac{a}{W}\right)}\;$$

(5) $$K_{r}=\frac{K_{I}}{K_{mat}},$$

(6) $$K_{I}=\sigma_{ref}\sqrt{\pi a\cdot sec\frac{\pi a}{2W}},$$

According to the carried out analysis, it can be concluded that the material of element A is more heavily loaded and has a smaller reserve of operational safety compared to element B. Comparing the loading of the specimens without a crack (*K*_r_ = 0), it can be noticed that the value of *L*_r_ for A is more than twice as high as for B. Although both specimens are in the safe area when loading the plates without a defect, the safety reserve for B is higher than for A. Assuming crack development in specimens of material A, the critical state will be reached for more shortly length crack than of B ones. The critical state of material A is reached for crack with dimensions *a*/*W* = 0.29 for the transverse specimen A_TL_ and *a*/*W* = 0.35 for the longitudinal one A**_LT_**, while for material B, it will only be reached for *a*/*W* = 0.74 and 0.79 for specimens B**_TL_** and B_LT_, respectively.

When specimens from materials *A* and *B* are loaded with the same stress level *σ*_ref_ = 130 MPa, we obtain similar results.

## 4. Discussion of the Results Obtained

This paper presents an assessment of the structural integrity of seaport portal crane elements A (jib) and B (boom) after their long-time operation of more than 33 years. Based on the results of the uniaxial tensile test, it was found that the yield strength values for the material taken from elements A and B in the longitudinal and transverse directions are similar. However, there is a clear difference in the *σ*_uts_ values and plasticity characteristics: the higher *σ*_uts_ values are for material A, while the higher values of the plasticity characteristics—for material B. Comparing the results obtained for specimens cut out in the longitudinal and transverse directions, it can be stated that slightly higher *σ*_uts_ and plasticity characteristics values were received for the longitudinal specimens compared to the transverse ones. The differences in the level of plasticity characteristics for longitudinal and transverse specimens were associated with the specific orientation of non-metallic inclusions in the plates along the rolling direction. This influence of non-metallic inclusions on the properties’ anisotropy is mainly revealed at the stage of neck formation in the specimens.

These elements are made of the same type of steel, but some differences in the microstructure were observed—the grain size in steel A is slightly larger than in B. The differences in grain size and different sheet thicknesses (16 mm for A and 12 mm for B) are caused by differences in thermo-mechanical processing during their production. For this reason, slightly higher values of plasticity characteristics *EL* (8–9% for specimen *LT* direction and 10–11%—for *TL* direction) and *RA* (7–7.2% for *LT* and 9.6–10.6% for *TL*) were observed in material B. The influence of grain size on the fracture toughness of the material is higher. In the presented studies, the value of fracture toughness *K*_JC_ of *LT* specimen direction is 17–20% higher for B compared to A and it is 12–14% higher for *TL* specimen direction. However, these results (plasticity and fracture toughness) are also influenced by non-metallic inclusions. However, it is not possible to separate the influence of grain size and inclusion particles.

Higher fracture toughness was obtained on the same oriented specimens taken from element B compared to element A. In specimens from element B, higher critical values of the integral *J*—*J*_C_ corresponding to the start of a subcritical crack were observed. On the other hand, the values characterising the development of the subcritical crack, tearing modulus—*T*_R_, are slightly higher for specimens from element B than from A for the appropriate orientation.

The FITNET-for-Service procedure from chapter Fracture was used for analysis. The analysis was performed at the basic (first) level according to the Failure Assessment Diagram (FAD) approach. In order to perform the evaluation according to the FAD approach, the strength, plasticity, and fracture toughness characteristics of the tested materials, A and B, were determined.

Comparing the results of FAD (FINTET) analysis for the studied elements, it can be concluded that at the current level of service loads, *σ*_ref_ = 130 MPa for element A and *σ*_ref_ = 55 MPa for B, and in the absence of the occurrence of crack-type defects, both elements are not at risk of failure due to the plasticisation of the section. The collapse ratio, calculated as *L* = *L*_r_max_/*L*_r_, is equal to: *L_A_*_L_ = 1.27/0.4 = 3.17 for A; *L_B_*_L_ = 1.22/0.16 = 7.62 for B. We obtain similar results when analysing the occurrence of load situations in the transverse direction. Similar conclusions are presented by the other authors who performed strength assessment studies of elements for similar portal cranes [23,34]. In a paper by M. Chen and S. He [36], FEM analysis data of a crane structure subjected to in-service loading was presented, and it was found that the highest equivalent stress level in the elements was equal to 131.3 MPa. In a study performed by O. Nemchuk, O. Nesterov et al. [23], the results of numerical calculations were supplemented by strain gauge measurements during the true operation of the crane at the locations where the highest values according to FEM calculations were obtained. On the basis of these tests, it was found that the maximum stress level in the structure reaches a value of up to 130 MPa, which is more than twice lower than the yield stress level (*σ*_YS_ = 280–300 MPa).

Carrying out the assessment according to the FAD method also allows the occurrence of a critical situation to be estimated, taking into account the presence of a crack in the element and its growth. In the analysis carried out on 1.0 m wide plates, it was shown that in the case of a central crack in the element at loads causing stress level *σ*_ref_ = for A and *σ*_ref_ = for B, the critical situation will be reached: in element A for *a*/*W* = 0.29 for orientation A_TL_ and *a*/*W* = 0.35 for A_LT_; in element B for *a*/*W* = 0.74 for orientation B_TL_ and *a*/*W* = 0.79 for B_LT_. The FAD methods of estimating the element conditions allow a comprehensive assessment to be carried out that simultaneously considers the effects of plasticity and fracture on the failure process of the element. In order to carry this out, experimentally determined strength and fracture toughness characteristics of the material of the component under test are necessary.

It should be emphasised that FITNET procedures were developed mainly based on API579/ASME-FFS [44], BS7910 [45], and R6 [46] standards. They are oriented towards the assessment of elements containing cracks, where the load is applied perpendicularly to the plane of the crack (Mode I). This does not exclude that these procedures cannot be applied during loading according to other schemes—mode II, III, or mixed. However, there are currently no established methods for determining *K*_r_ and *L*_r_ during mixed loading modes. These quantities can be determined at relatively low load levels where the principles of linear-elastic fracture mechanics can be used. Such a situation occurs during fatigue crack propagation, in the threshold region of the *V* = *f*(Δ*K*_I_) relationship (Region I) and in the lower part of the region described by Paris’s law (Region II). Therefore, in the case of mixed loading, it was proposed to use *K*_eq_ instead of *K*_I_. The analysis of the application of mode I and II loads to describe the development of fatigue cracks according to several models is presented in [47]. The comparison of the results indicates that the modelled values exceed the experimental ones by about 10–40%, depending on the model used.

## 5. Conclusions

The article presents the results of strength and fracture toughness tests of two elements A (jib) and B (boom) of the portal crane after long-term (for about 33 years) operation, and their structural integrity assessment based on FAD (FITNET) Analysis. The results suggested the following:

1. It has been shown that the level of strength characteristics is slightly higher in element A. On the other hand, the plasticity and fracture toughness characteristics in the material of element B are higher than in A. The reason for this may be the finer grains in the microstructure of material B and the lower level of operating stresses.
2. Higher fracture toughness was obtained on the same oriented specimens taken from element B compared to element A. Fracture toughness *J_C_* is characterised by a significantly higher anisotropy compared to the basic tensile properties. Also, as shown in previous studies [25], the impact toughness characteristic is very sensitive to the microstructural details of the rolled steel.
3. The results of the FAD (FITNET) analysis performed for the elements containing a central crack loaded by tensile stress showed that they are not susceptible to failure. The relative length of cracks for which the critical state will be reached is also estimated: in element A for *a*/*W* = 0.29 for orientation A_TL_ and *a*/*W* = 0.35 for A_LT_; in element B for *a*/*W* = 0.74 for orientation B_TL_ and *a*/*W* = 0.79 for B_LT_.

## Figures and Tables

**Table 1:** Chemical composition of the analysed steel (% by weight) [25].

C	Si	Mn	Cr	S	P	Cu	Fe
0.17	0.23	0.54	0.11	0.01	0.01	0.1	in balance

**Table 2:** Strain and plasticity characteristics of the specimens cut out of A and B elements.

Scheme	*σ*_LYS_ [MPa]	*σ*_HYS_ [MPa]	*σ*_UTS_ [MPa]	*EL*_U_ + *EL*_NU_ [%]	*EL*_F_ [%]	*R*A [%]
A_LT_ (Longitudinal)	281.7	299.6	442.2	18.7 + 13.2	31.9	76.4
A_TL_ (Transverse)	284.1	296.5	437.4	15.9 + 14.6	30.5	70.6
B_LT_ (Longitudinal)	287.5	305.6	417.1	20.9 + 13.8	34.7	82.3
B_TL_ (Transverse)	283.1	299.1	411.5	18.9 + 14.9	33.8	78.1

**Table 3:** The fracture toughness characteristics of the specimens cut out of elements A and B.

Crane Unit	Specimen Code	*J*_C_ [KN/m]	*K*_JC_ [MPa∙m^1/2^]	*dJ*/*da* [MPa]	*T*_R_	*KCV* [J/cm^2^]
Jib	A_LT_	173.7	195.4	230	511.1	159
	A_TL_	123.3	164.6	108	245.7	60
Boom	B_LT_	250.9	234.8	234	514.2	310
	B_TL_	159.5	187.2	134	298.6	123

**Table 4:** Fracture toughness values used for elements structural integrity assessment FAD procedure.

Specimen Thickness	Fracture Toughness		Specimen Orientation			
			A_LT_	A_TL_	B_LT_	B_TL_
*B* = 12 (mm)	*K*_JC_	(MPa$\sqrt{m}$)	195.4	164.6	234.8	187.2
*B* = 25 (mm)	*K*_mat_	(MPa$\sqrt{m}$)	166.0	140.4	198.8	159.2

## Data Availability

The original contributions presented in this study are included in the article. Further inquiries can be directed to the corresponding author.
